# Infective endocarditis in the Lao PDR: Clinical characteristics and outcomes in a developing country

**DOI:** 10.1016/j.ijcard.2014.11.184

**Published:** 2015-02-01

**Authors:** Mariana Mirabel, Sayaphet Rattanavong, Khamthavy Frichitthavong, Vang Chu, Pany Kesone, Phonvilay Thongsith, Xavier Jouven, Pierre-Edouard Fournier, David A.B. Dance, Paul N. Newton

**Affiliations:** a INSERM U970, Paris Cardiovascular Research Center PARCC, Paris, France; b Université Paris Descartes, Sorbonne Paris Cité, Paris, France; c Assistance Publique-Hôpitaux de Paris, Hôpital Européen Georges Pompidou, Paris, France; d Lao-Oxford-Mahosot Hospital-Wellcome Trust Research Unit (LOMWRU), Microbiology Laboratory, Mahosot Hospital, Vientiane, Lao People’s Democratic Republic; e Cardiology Department, Mahosot Hospital, Vientiane, Lao People’s Democratic Republic; f Unité de Recherche sur les Maladies Infectieuses et Tropicales Emergentes, Centre National de la Recherche Scientifique–Institut de Recherche pour le Développement, Unité Mixte de Recherche 6236, Faculté de Médecine, Université de la Méditerranée, France; g Centre for Tropical Medicine and Global Health, Nuffield Department of Clinical Medicine, Churchill Hospital, University of Oxford, Oxford, United Kingdom

**Keywords:** Infective endocarditis, Rheumatic heart disease, Septicemia, Heart valve disease, Developing countries, Laos, Lao PDR

## Abstract

**Introduction:**

Data on infective endocarditis (IE) in Southeast Asia are scarce.

**Objectives:**

To describe the clinical epidemiology of IE in Lao PDR, a lower middle-income country.

**Methods:**

A single centre retrospective study at Mahosot Hospital, Vientiane. Patients aged over 1 year of age admitted 2006–2012 to Mahosot Hospital with definite or possible IE by modified Duke criteria were included.

**Results:**

Thirty-six patients fulfilled the inclusion criteria; 33 (91.7%) had left-sided IE. Eleven (30.6%) had definite IE and 25 (69.4%) possible left-sided IE. Median age was 25 years old [IQR 18–42]. Fifteen patients (41.7%) were males. Underlying heart diseases included: rheumatic valve disease in 12 (33.3%), congenital heart disease in 7 (19.4%), degenerative valve disease in 3 (8.3%), and of unknown origin in 14 (38.9%) patients. Native valve IE was present in 30 patients (83.3%), and prosthetic valve IE in 6 patients (16.7%). The most frequent pathogens were *Streptococcus* spp. in 7 (19.4%). Blood cultures were negative in 22 patients (61.1%). Complications included: heart failure in 11 (30.6%), severe valve regurgitation in 7 (19.4%); neurological event in 7 (19.4%); septic shock or severe sepsis in 5 (13.9%); and cardiogenic shock in 3 patients (8.3%). No patient underwent heart surgery. Fourteen (38.9%) had died by follow-up after a median of 2.1 years [IQR 1–3.2]; and 3 (8.3%) were lost to follow-up.

**Conclusions:**

Infective endocarditis, a disease especially of young adults and mainly caused by *Streptococcus* spp., was associated with rheumatic heart disease and had high mortality in Laos.

## Highlights

• Data on infective endocarditis (IE) in Southeast Asia are scarce. Most reports on IE originate from tertiary centres in Europe and North America and may not reflect the world's epidemiology of IE.
• In Laos, IE remains a disease of young adults with no comorbidities, mainly caused by *Streptococcus* spp., associated with rheumatic heart disease.
• Access to imaging techniques is limited and over half the patients have negative blood cultures.
• Cardiac surgery is not routinely used in Laos for acute IE.
• Mortality at 2 years follow-up is unacceptably high reaching 39%.

## 1 Introduction

Infective endocarditis (IE) is a rare but severe disease that still has a high mortality, even in those with access to tertiary centres. The epidemiology of IE has recently significantly changed across North America and Europe in affecting an increasingly ageing population with comorbidities. Presentation is nowadays often acute, characterized by high rates of *Staphylococcus aureus* infection, cardiac complications, and embolic events [1–3]. Guidelines for prevention and management of IE are based on Western-focused studies, with an increasing emphasis on early heart surgery [4–7]. However, data from developing countries are scarce [8–11]. Driven by other epidemiological characteristics, the challenges and treatment options the physician encounters in middle and low-income settings may differ greatly from those described in the medical literature.

We describe the clinical characteristics of IE in patients admitted to a tertiary teaching hospital, in Vientiane, the Lao PDR (Laos), a lower middle-income country, and examined their long-term outcomes.

## 2 Methods

### 2.1 Objectives

The main objective was to describe the characteristics of patients admitted with IE to a tertiary centre in Laos, a lower middle-income country.

### 2.2 Study population, study site and microbiological tests

Patients aged over 1 year admitted from January 2006 to January 2012 to Mahosot Hospital with definite or possible IE according to the modified Duke criteria were included in the study [12]. Mahosot Hospital (17.960 N, 102.612 E), Vientiane, is a primary–tertiary care teaching hospital, with ~ 400 beds including cardiology wards that provide cardiac surgery, mostly by visiting teams, and infectious disease wards. The hospital has trans-thoracic echocardiography; trans-oesophageal echocardiography is used by visiting surgical teams. Blood cultures were performed using standard procedures and antibiotic susceptibility patterns determined using Clinical and Laboratory Standards Institute (CLSI) methods [13]. The clinical significance of positive cultures was determined by a team of physicians at the time of the result based on factors that included organism identity and the number of samples growing the same organism. We tested (acute and convalescent) sera for antibodies to *Coxiella burnetii*, *Legionella pneumophila*, *Bartonella quintana and Bartonella henselae* by indirect immunofluorescence assay (IFA) as previously described [14]. Specific antibodies to *Brucella melitensis* and *Mycoplasma pneumoniae* were detected with an immunoenzymatic antibody test and the Platellia *M. pneumoniae* IgM kit (Bio-Rad, Marnes-la-Coquette), respectively. When the results of the tests described above were negative, we performed Western blot using *Bartonella* species antigens, as described [15,16].

Informed consent was given by the patient or the next of kin at the time of the blood culture as part of a study of the aetiology of septicaemia. Ethical clearance was provided by the Ethical Review Committee of the Faculty of Medical Sciences, National University of Laos (Vientiane, Laos) and the Oxford University Tropical Ethics Research Committee (Oxford, United Kingdom). The authors of this manuscript have all certified that they comply with the Principles of Ethical Publishing.

### 2.3 Data collection

The hospital charts of patients with a clinical diagnosis or suspicion of IE were retrospectively reviewed by two of the authors (MM and SR). Patients were identified through the Echocardiography Laboratory logbooks and the blood culture database of the Microbiology Laboratory. For each patient, the following data were collected: demographics; symptoms and signs, including details of comorbidities such as diabetes, treatment with steroids, excess alcohol consumption, IV drug use, and HIV infection. Supplementary data were collected through the hospital charts and the echocardiography logbooks: heart valve disease and its aetiology; history of cardiac surgery; IE complications as severe valve dysfunction, heart failure (including cardiogenic shock), septic shock or severe sepsis, neurological complication, and arterial embolism. Definitions of IE complications were based on contemporary guidelines [4]. The type of echocardiography (transthoracic, transoesophageal or both), valve involved, vegetation detected and its maximum length were recorded. Patients with both left- and right-sided IE were assigned to the left-sided group. Those with native valve IE and prosthetic valve IE were assigned to the latter. The choice of valve affected was based upon the presence of vegetation, abscess and/or fistula on echocardiogram. When only transthoracic echocardiogram was performed and none of these structures were described, the choice of the valve affected was based upon the presence of at least moderate valve disease or a prosthetic valve.

Outcomes measures were: in-hospital mortality, status when discharged (alive, unwell, or moribund), relapse, and long-term mortality. Follow-up was undertaken as a cross sectional study by contacting the patient and/or his/her next of kin. If the patient was lost to follow-up, the date of the latest attendance to the clinic was recorded.

## 3 Statistical analyses

The results are reported as median and interquartile range (IQR) or as numbers and percentages. Categorical variables were compared using chi-square test or Fisher's exact test, and continuous variables using Student t-test or Wilcoxon rank sum test, as appropriate. Significance was defined as p-values less than 0.05. Statistical analyses were performed using STATA SE/12.1 (StataCorp LP, College Station, TX).

## 4 Results

After review of 110 patients' hospital charts, 36 fulfilled the modified Duke criteria for IE and were included in the study; 11 (30.6%) had definite IE and 25 (69.4%) possible IE (Table 1). Median age was 25 years old [IQR 18–42]. Fifteen patients (41.7%) were males. Thirty-three out of 36 patients (91.7%) had left-sided IE. Five (13.9%) patients were diabetic, 2 (5.6%) were on steroids, and 1 (2.8%) admitted excessive alcohol consumption. No patient was on chronic replacement renal therapy. No IV drug users or HIV positive patients were identified. Underlying heart diseases included rheumatic valve disease in 12 (33.3%), congenital heart disease in 7 (19.4%) and degenerative valve disease in 3 (8.3%) patients. Eight (22.2%) patients had no evidence of underlying valve disease, and data was missing for 6 (16.7%). Infective endocarditis afflicted a prosthetic valve in 6 (16.7%) patients. The presence of a vegetation, abscess or fistula allowed to formally diagnose mitral valve IE in 11 (30.6%), aortic valve IE in 9 (25%) and both the mitral and aortic valves in 2 patients (5.6%). Further 5 patients were diagnosed with mitral, 2 with aortic and mitral valve, and one with aortic valve IE based on the severity of valve regurgitation or the presence of a prosthetic valve, giving a total of 16 (44.4%) mitral, 10 (27.8%) aortic valve, and 4 (11.1%) combined aortic and mitral valve IE. One patient (2.8%) had unrepaired ventricular septal defect (VSD) IE with vegetations both on the VSD and the tricuspid valve. One patient had unrepaired tetralogy of Fallot with no vegetation and was diagnosed with left-sided IE. One patient had mild mitral regurgitation with a neurological defect but no vegetation on TTE and was classified as having left-sided IE. One patient presented with vegetation on pulmonary ductus arteriosus. Two patients did not undergo TTE during their hospital stay.

Investigations included TTE in 34 (94.4%) patients. All patients had at least one set of blood cultures. One (2.8%) patient underwent brain CT scan. None underwent transoesophageal examination.

At the date when blood cultures were drawn, symptoms had been present for 7 days (median [IQR 2–21]). Blood cultures were negative in 22 out of 36 patients (61.1%, missing data in one case), of whom 4 had definite IE and 18 possible IE. One patient with blood culture negative IE had evidence for *B. henselae* infection. Pathogens were isolated on blood cultures from 13 (36.1%) patients: *Streptococcus* spp. in 7/13 (*Streptococcus pyogenes* in 2, *Streptococcus oralis* in 1, *Streptococcus mutans* in 1, *Streptococcus anginosus* in 1, *Streptococcus sanguinis* in 1 and *Streptococcus agalactiae* in 1), followed by *Escherichia coli* in 2/13, *Enterococcus faecalis* in 2/13, *Staphylococcus aureus* in 1/13 (2.7%), and coagulase negative staphylococci in 1/13 (in a patient with a prosthetic aortic valve). Serology gave no evidence for *C. burnetii*, *L. pneumophila*, *B. melitensis* or *M. pneumoniae* infection in those with negative blood cultures. The antimicrobial susceptibilities of the organisms isolated were largely as expected for the species concerned. All seven *Streptococcus* spp. tested for their penicillin minimum inhibitory concentration (MIC) by Etest (bioMérieux, Basingstoke, UK) were fully susceptible (MIC ≤ 0.125 mg/L). Both *E. coli* isolates were fully susceptible to ceftriaxone and gentamicin by disk diffusion testing although both were resistant to ampicillin. Both *E. faecalis* isolates were susceptible to penicillin or ampicillin (MIC ≤ 4 mg/L) although one showed evidence of high level resistance to gentamicin. Both *Staphylococcus* spp. had oxacillin MICs < 1 mg/L.

Clinical complications during hospital stay included: heart failure with no shock in 11 (30.6%); severe valve regurgitation in 7 (19.4%); neurological complication in 7 (19.4%); septic shock or severe sepsis in 5 (13.9%); and cardiogenic shock in 3 (8.3%) patients. Among the 11 patients with detailed echocardiography reports, valve obstruction was noted in 4/11; and 2/11 presented with large vegetations (i.e. > 10 mm as the longest dimension), abscess or fistula on TTE.

The initial antibiotic regimen given was available for 25 out of 36 patients, among whom 10 received a combination of ceftriaxone and gentamicin; 5 ceftriaxone alone; 4 a combination of penicillin and gentamicin; 1 a combination of ampicillin and gentamicin; 1 penicillin alone; 1 cloxacillin alone; 1 a combination of cloxacillin and gentamicin; 1 a combination of cloxacillin and rifampicin; and 1 a combination of ceftriaxone, gentamicin and doxycycline.

No patient underwent surgery either during the acute phase or after completion of antibiotic treatment. The median length of hospital stay was 16 days [IQR 9–21].

Nine (25.0%) patients died during their hospital stay whilst 14 (38.9%) had died by follow-up after a median of 2.1 years [IQR 1–3.2]. Three (8.3%) patients were lost to follow-up. Among the 10 patients discharged unwell, 6 were dead at follow-up, 3 were alive, and 1 was lost to follow-up. Five patients (13.9%) experienced IE relapse requiring readmission. Causes of death were available in 6 out of 14 cases: heart failure in 3 patients; shock in 1 patient; gastrointestinal bleed in 1 case; and ventricular fibrillation and acute renal failure in the remaining patient. The only factor associated with long-term mortality on univariate analysis was greater age (p = 0.02) (Table 2).

## 5 Discussion

We describe the first series of patients with IE in Laos. Patients were mostly young with mainly rheumatic heart disease as their underlying condition and rare comorbidity. The main causative agents were *Streptococcus* spp., but blood cultures remained negative in over half the patients. No patient underwent transoesophageal echocardiography as a routine diagnostic tool. Hospital stay was relatively short and the rate of relapses was high. Due to lack of resident cardiac surgeons, surgery could not be performed in the presence of clear indications such as heart failure or cardiogenic shock. Mortality was extremely high, reaching ~ 39% at 2 years follow-up.

These data highlight the marked differences in IE epidemiology between developing and wealthy countries. The demographic characteristics, high frequency of underlying rheumatic heart disease and microbiological aetiology are consistent with reports from other developing countries [9,17–20,36].

Rheumatic heart disease, a neglected disease of poverty, remains the most common underlying condition. However, prevention programmes reduce the burden of the disease. Control of rheumatic heart disease can be achieved through cost-effective, register-based, primary and secondary prevention programmes that deliver penicillin [21]. Congenital heart disease was the second most frequent underlying aetiology, probably because of the inclusion of children in our series.

The high frequency of streptococcal species in the Lao patients probably in part reflects poor oral health [22], suggesting the interest of promoting dental hygiene education and awareness. Over half the patients had negative blood cultures. This finding is consistent with other series from developing countries [8,9,11,19]. Over the counter antibiotics are available in Laos and self-medication is common [23], and may have contributed to the low frequency of positive blood cultures, although the retrospective design of our study did not allow this to be assessed. Some of the pathogens identified, such as *E. coli* and *S. pyogenes*, have been described as causing IE but are rare in industrialised country case series [24,25]. On the basis of the results of this series, initial empirical treatment of patients with suspected endocarditis with ceftriaxone and gentamicin would be appropriate in Laos, adjusted on the basis of susceptibilities if an organism is grown.

Access to imaging techniques was relatively low, with no transoesophageal echocardiography performed. Only a minority of patients (1 out of 7) with clinical neurological complications underwent a brain CT scan. Contrary to international guidelines [4,5], cephalosporins were frequently the first line of treatment, because of the logistic difficulties of providing repeated infusions of penicillin. Median length of stay was 16 days, suggesting that many patients did not receive a minimum of 14 days of intravenous treatment, likely responsible for the high rate of relapses (13.9%).

Access to cardiac surgery remains limited in Laos. No patient in this series underwent cardiac surgery either during acute IE or after completion of antibiotic treatment.

Outcomes were poor with approximately 39% of patients dead at follow-up. These findings are worrying considering the clinical characteristics of the disease: young patients with streptococcal infections, and barely any comorbid conditions. Indeed, viridans streptococcal IE has been documented as having a good prognosis when compared to other causative microorganisms [1]. Younger age also should be associated with higher chances of survival according to other series [1,26]. The deadly toll in this series is consistent with reports from other developing countries [18,19,27,28].

In contrast to our findings, Thuny and colleagues recently outlined the tremendous progress achieved in the management of IE in wealthy countries [7]. Mortality is as low as 8.2% in some high-volume centres [29]. Early surgery has become a mainstay in the therapy of complicated IE [4,5,30]. Such improvement is nonetheless largely restricted to the wealthy world. In many developing countries, limited access to health facilities, lack of adequate diagnostic tools such as transthoracic and transoesophageal echocardiography, inadequate laboratory facilities to perform trustworthy blood cultures and susceptibility testing and insufficient skilled staff hamper early reliable diagnoses. Antibiotics are not necessarily appropriate or of good quality [23,31]. The treatment course is commonly interrupted prematurely as patients cannot afford the parenteral antibiotics, inpatient charges and family income loss associated with prolonged hospitalisation. These results suggest the need for prospective studies assessing reasons for early discharge in developing countries and what engagement strategies may help to reduce this. Importantly, access to cardiac surgery is limited in most low- and lower-middle income countries. For instance, surgical facilities are restricted to only 15 out of 55 African states, often supported by visiting teams from Europe or North America [32].

Prevention of rheumatic heart disease and access to dental health services may reduce the incidence of IE worldwide. Considerable investment in national referral centres for access to diagnosis, inexpensive, good quality, antibiotics and resources to support patients with long term therapy and offset income loss are likely to be vital in reducing the burden of this curable neglected infectious disease.

## 6 Strength and limitations

This study is one of the very few reports on IE from Southeast Asian developing countries [18,33–35]. It provides valuable data on the clinical characteristics of IE in Laos. Although retrospective, follow-up data was available in the majority of patients providing long-term mortality rates. Access to diagnostic techniques was limited hence patients with possible IE were included in the study. This study has a number of limitations. The sample size was relatively small and therefore predictors of mortality could not be thoroughly assessed. The inclusion of children in our series may have overrepresented, in comparison to series of only adults, congenital heart disease as an underlying cause of IE. We cannot rule out the possibility of a referral bias. Visiting surgical teams from Europe (Luxembourg and France) have been working at our institution on a regular basis for the past 10 years with an emphasis on curable congenital defects.

## 7 Conclusion

Infective endocarditis has a high mortality rate in Laos, especially afflicting young adults with rheumatic heart disease and mainly caused by *Streptococcus* spp. Ways to reduce the burden of disease through rheumatic heart disease prevention, promotion of oral health, and treatment options by increasing diagnostic and therapeutic capacities need to be addressed in developing countries.

## Conflict of Interest

No conflict of interest to declare.

## Figures and Tables

**Table 1:** Diagnostic criteria in 36 patients with definite or possible IE.

Typical organism in 2 separate cultures^a^, n^f^ (%)	9 (25.0)
Persistently positive blood cultures^b^, n (%)	0
Positive blood culture that does not meet the major criteria	4 (11.1)
Echocardiogram positive for IE^c^, n (%)	24^g^ (66.7)
New valvular regurgitation, n (%)	1 (2.8)
Predisposing heart condition, n (%)	24 (66.7)
Fever > 38 °C, n (%)	31 (86.1)
Vascular phenomena^d^, n (%)	10 (27.8)
Immunological phenomena^e^, n (%)	1 (2.8)

a Including *Streptococcus viridans*, *Streptococcus bovis*, HACEK group, *Staphylococcus aureus* or community-acquired enterococci.

b Defined as microorganisms consistent with IE from persistently positive blood cultures.

c Defined as one of the following: oscillating intracardiac mass on valve or supporting structures, in the path of regurgitant jets, or on implanted material in the absence of an alternative anatomic explanation; or abscess.

d Defined as: major arterial emboli, septic pulmonary infarcts, mycotic aneurysm, intracranial haemorrhage, conjunctival haemorrhages, and Janeway's lesions.

e Defined as glomerulonephritis, Osler's nodes, Roth's spots, and rheumatoid factor.

f Missing data in one case.

g One patient had perivalvular abscess with no vegetation seen. No. patient underwent heart valve surgery or necropsy precluding fulfilment of pathological criteria.

**Table 2:** Clinical, echocardiographic and microbiological characteristics according to long-term mortality.

Factor	Alive at follow-up n = 19	Dead at follow-up n = 14
Age years, mean (SD)	28.4 (3.1)	42.8 (5.4)
Male, n (%)	7 (19.4)	6 (42.9)
SBP mm Hg, mean (SD)	103.3 (4.6)	108.2 (8.2)
HR pulse/min, mean (SD)	102.1 (5.6)	96.4 (4.9)
Temperature °C, mean (SD)	38.5 (1.1)	38.1 (0.9)
Definite IE, n (%)	5 (26.3)	5 (13.9)
NVE, n (%)	15 (78.9)	13 (92.9)
Streptococcal IE, n (%)	2 (10.5)	3 (21.4)
Rheumatic heart disease, n (%)	7 (72.6)	5 (35.7)
Complicated IE^a^, n (%)	7 (36.8)	9 (64.3)
Relapse IE, n (%)	3 (15.8)	1 (7.1)
Vegetation on TTE, n (%)	13 (68.4)	10 (71.4)
Discharged unwell, n (%)	3 (15.8)	6 (42.9)

SBP, systolic blood pressure mm Hg. HR, heart rate/min. NVE, native valve endocarditis.

a Defined by one of the following: severe valve regurgitation, septic shock or severe sepsis, heart failure with no shock, cardiogenic shock, neurological complication, valve obstruction, large vegetations (i.e., > 10 mm), abscess or fistula.
